# Relation Between Neutrophil Count and Left Ventricular Ejection Fraction Following Acute Myocarditis in Adolescents: A Preliminary Study

**DOI:** 10.3390/children13010040

**Published:** 2025-12-27

**Authors:** Barbara Rabiega, Dominika Wysocka, Tomasz Urbanowicz, Anna Olasińska-Wiśniewska, Marek Jemielity, Waldemar Bobkowski

**Affiliations:** 1 Pediatric Cardiology Department, Poznan University of Medical Sciences, 60-572 Poznan, Poland; dwysocka@skp.ump.edu.pl (D.W.);; 2 Cardiac Surgery and Transplantology Department, Poznan University of Medical Sciences, 61-848 Poznan, Poland

**Keywords:** myocarditis, neutrophil, pediatric cardiology, left ventricular ejection fraction improvement

## Abstract

(1) Background: The clinical course of acute myocarditis in adolescents is heterogeneous, and reliable predictors of early functional changes remain limited, particularly in patients without severe systolic dysfunction. Routine hematologic parameters may reflect the early inflammatory response, but their prognostic relevance in pediatric non-fulminant myocarditis is poorly defined. This exploratory study aimed to assess whether admission inflammatory blood indices are associated with short-term changes in left ventricular systolic function in adolescents with acute myocarditis. (2) Methods: We retrospectively analyzed 44 adolescents (median age 16 years, 84% male) hospitalized with suspected acute non-fulminant myocarditis between 2020 and 2023. All patients had preserved or mildly reduced left ventricular ejection fraction (LVEF) at presentation. Clinical, laboratory, electrocardiographic, and echocardiographic data obtained at admission were analyzed. Changes in LVEF between the acute and post-acute phases during hospitalization were assessed using transthoracic echocardiography. Cardiac magnetic resonance imaging was performed in a subset of patients to support diagnosis but was not uniformly available for quantitative analysis. (3) Results: No in-hospital deaths occurred. A modest positive correlation was observed between neutrophil count at admission and improvement in LVEF during hospitalization (r = 0.348, *p* = 0.028). No significant associations were found between LVEF change and white blood cell count, lymphocyte count, monocyte count, neutrophil-to-lymphocyte ratio (NLR), troponin I, or NT-proBNP. (4) Conclusions: In adolescents with non-fulminant acute myocarditis and preserved or mildly reduced systolic function, admission neutrophil count was associated with short-term improvement in left ventricular ejection fraction. Given the retrospective design, limited sample size, and absence of mechanistic data, these findings should be interpreted as hypothesis-generating. Further prospective studies incorporating standardized cardiac magnetic resonance imaging and immunologic profiling are needed to clarify the clinical significance of this association.

## 1. Introduction

Myocarditis in the pediatric population remains a clinical challenge due to its heterogeneous clinical presentation, variable biomarker elevation, and often mild initial symptoms [1]. Historically, a substantial proportion of myocarditis diagnoses were established post mortem [2]. The wide spectrum of symptoms and abnormalities makes diagnostics difficult [3]. Gender differences have been observed in the young population, with a predominance of male patients [4]. Although the reported mortality rate is relatively high (<10%), first-line therapy is guided by clinical presentation, as the disease course is uncomplicated in approximately 75% of patients [5]. Nevertheless, acute myocarditis may rapidly progress to a fulminant form accompanied by heart failure and hemodynamic instability [6]. Such severe phenotypes were excluded from the present analysis.

According to current recommendations, a multimodal diagnostic approach is advised when myocarditis is suspected. Typical diagnostic findings include electrocardiographic (ECG) abnormalities such as ST–T segment changes, low QRS voltage (which may reflect concomitant pericardial effusion), atrioventricular block, QT prolongation, and various cardiac arrhythmias [2]. Some patients require antiarrhythmic therapy or the implantation of a temporary or permanent pacemaker [7]. In the pediatric population, current guidelines emphasize the importance of arrhythmia surveillance (including 24-h Holter monitoring, resting ECG, and exercise ECG) during the recovery period—even in patients with preserved LVEF—due to the potential risk of electrical complications [8].

Imaging studies are also essential. Echocardiography (ECHO) remains the first-line modality for assessing ventricular systolic function when myocarditis is suspected [2]. Typical ECHO findings include reduced LVEF, pericardial effusion, increased left ventricular dimensions, and valvular regurgitation [9].

Cardiac magnetic resonance imaging (CMR) is utilized to evaluate myocardial inflammation, edema, and necrosis [10].

Laboratory testing often reveals elevated inflammatory markers (C-reactive protein, leukocyte count) and cardiac injury biomarkers, including brain natriuretic peptide (BNP), N-terminal prohormone of brain natriuretic peptide (NT-proBNP), and troponin I; however, normal values do not exclude myocarditis [10].

Current guidelines also recommend periodic assessment of left ventricular function through repeated ECHO evaluations, serial biomarker monitoring, and ongoing clinical follow-up during the convalescent phase [11].

This integrative approach, combining biomarkers, imaging modalities, and clinical monitoring, constitutes the foundation for longitudinal diagnosis and management of myocarditis in clinical practice.

A viral infection typically precedes the onset of myocarditis, and cardiovascular involvement is primarily driven by immunological mechanisms [12,13]. The most common viral causes include adenoviruses and enteroviruses (coxsackie A, coxsackie B, and echoviruses); parvovirus B19; human herpesvirus 6 (HHV-6), Epstein–Barr virus (EBV), and cytomegalovirus (CMV); human immunodeficiency virus (HIV); hepatitis C virus (HCV); influenza A and B viruses; and severe acute respiratory syndrome coronavirus (SARS-CoV) [14]. Despite broad diagnostic capabilities, the etiology of myocarditis often remains unidentified.

Fulminant myocarditis represents a distinct and severe form of the disease, characterized by a marked reduction in LVEF, rapid hemodynamic deterioration, and high morbidity, often necessitating mechanical circulatory support [15,16]. However, even patients with preserved left ventricular contractility during acute myocarditis may face an increased risk of life-threatening complications [17]. In young individuals, even a mild reduction in LVEF may have clinically significant long-term consequences.

Viral infection and subsequent lysis of infected cardiomyocytes may trigger activation of both innate and adaptive immune pathways, which in physiological conditions contribute to inflammation resolution and myocardial improvement. In a subset of patients, however, the inflammatory response becomes persists, resulting in permanent impairment of left ventricular systolic function or progression to adverse clinical phenotypes, including dilated cardiomyopathy, acute and chronic heart failure, and ventricular arrhythmias [18].

Management of myocarditis remains predominantly symptomatic. Current recommendations include bed rest and restriction of physical activity. Pharmacotherapy, such as diuretics, angiotensin-converting enzyme inhibitors, vasodilators, or inotropes, is administered according to clinical need [19]. Importantly, no established therapeutic intervention directly targets or eliminates the underlying inflammatory trigger.

Inflammatory markers assessed through complete blood count have prognostic relevance across diverse clinical conditions, including cardiovascular, infectious, oncologic, and gastrointestinal diseases [20]. Neutrophils play a crucial role in the initial inflammatory response [21].

Produced in the bone marrow, only fully mature neutrophils enter the circulation. Their recruitment and activation are rapidly induced by microbial invasion or tissue injury [22]. Once migrated to affected tissues, neutrophils initiate immediate antimicrobial activity. Studies evidence suggests that appropriately regulated neutrophil recruitment contributes to clearance of necrotic debris and facilitates subsequent reparative processes [23].

Despite their protective functions, neutrophils may also exert cytotoxic effects on myocardial tissue by releasing reactive oxygen species, proteolytic enzymes, and proinflammatory mediators [24,25].

In a murine model of Coxsackievirus B3–induced viral myocarditis, early inhibition of neutrophil activity and neutrophil extracellular traps (NET) formation was shown to attenuate myocardial necrosis and inflammation, primarily by reducing monocyte recruitment and the activation of pro-inflammatory macrophages. This protective effect was limited to the acute phase only. Inhibition of neutrophil activity after the acute phase does not improve the course of myocarditis [26].

Müller et al. [27] demonstrated that circulating S100A8/A9—an alarmin complex released predominantly by activated monocytes, macrophages, and neutrophils—is significantly elevated in both acute and chronic myocarditis compared with non-inflammatory heart failure. S100A8/A9 levels correlated with established inflammatory and myocardial injury biomarkers, including troponin and NT-proBNP. This suggests that S100A8/A9 may serve as a biomarker of inflammatory activity in myocarditis, reflecting the severity of the immune response [27].

In a study by Ichimura et al. [28], the authors showed that NETs are present in myocardial tissue and are associated with worse systolic function and adverse cardiac remodeling in patients with dilated cardiomyopathy/heart failure states. Their data suggest that NET burden in the myocardium may contribute to cardiac dysfunction and could represent a therapeutic target in heart failure settings [28].

In a multicenter study including 1150 patients with acute myocarditis, an elevated NLR ≥ 4 at presentation independently predicted a more than threefold increase in all-cause mortality or heart transplantation during follow-up, even among individuals presenting with preserved LVEF. This highlights the potential of NLR as a risk-stratification tool in ostensibly lower-risk myocarditis populations [29].

Given the variability in clinical presentation and outcomes, further research is warranted to identify accessible prognostic markers that can reliably predict adverse outcomes, facilitate early risk stratification, and guide clinical decision-making. Patients at high risk of progression to heart failure require prompt therapeutic intervention [30] and long-term follow-up.

Neutrophil count has also been identified as an independent predictor of reduced LVEF in patients with acute myocardial infarction, as reported by Arakawa et al. [31].

Inflammatory pathways in myocarditis have been studied in adult populations; however, there is limited understanding of the prognostic significance of routine hematologic markers, such as neutrophils and the NLR, in adolescents with non-severe myocarditis. It remains unclear whether neutrophil count reflects myocardial inflammation or correlates with subsequent left ventricular improvement.

The aim of this exploratory, retrospective study was to assess whether routinely available clinical, laboratory, electrocardiographic, and echocardiographic parameters obtained at hospital admission are associated with early changes in left ventricular systolic function in adolescents hospitalized with non-fulminant acute myocarditis. In particular, we focused on peripheral blood inflammatory markers, including absolute neutrophil count, as potential correlates of post-acute functional improvement. The aim of the study was to generate hypotheses regarding easily accessible prognostic indicators in a pediatric population with preserved or mildly reduced left ventricular ejection fraction.

## 2. Materials and Methods

Forty-four patients (37 (84%) male and 7 (16%) female), with a mean age of 16 (14–17) years, were admitted to the Pediatric Cardiology Department at Poznan University of Medical Sciences between January 2020 and December 2023 with suspected acute myocarditis and were enrolled in this retrospective analysis.

Patients with fulminant myocarditis, cardiac tamponade requiring surgical drainage, or Kawasaki disease were excluded from the study to avoid confounding influences on neutrophil counts and outcomes [32].

The etiology of myocarditis was infectious; no patients had a previous medical history of systemic disease, autoimmune disorders, or toxic myocarditis.

The diagnosis of acute myocarditis was established based on a combination of clinical presentation, laboratory findings, ECG abnormalities, and imaging studies, in accordance with contemporary recommendations.

Blood samples were collected on admission from all patients, and ECG and transthoracic ECHO exams were performed (on admission and in post-acute phase, on the day of discharge home).

CMR was performed in a subset of patients to support the diagnosis of myocarditis, depending on clinical stability, availability, and individual contraindications. Due to the retrospective design and non-uniform application of CMR, tissue characterization findings were not included in the correlation analyses. Echocardiography remained the primary modality for serial assessment of left ventricular systolic function during hospitalization. Endomyocardial biopsy is not routinely performed in pediatric populations [33].

During hospitalization, all patients were treated according to current guidelines [19]. Bed rest and activity restriction were recommended for all patients; and treatment with diuretics, angiotensin-converting enzyme inhibitors, vasodilators, and inotropes was used as necessary.

Patients were divided into two groups based on changes in LVEF during the course of the myocarditis, as assessed by ECHO. Group 1 consisted of patients with normal and unchanged LVEF during the episode (min > 67%, median 71%) and after the episode (median 71%), while group 2 consisted of adolescents who demonstrated mildly reduced LVEF during the acute phase (min < 67%, median 63%), with subsequent improvement toward normal values in the post-acute period (median 72%). Importantly, no patient in either group presented with severe systolic dysfunction, and left ventricular ejection fraction remained within the normal or mildly reduced range throughout hospitalization.

The median LVEF in group 1 was 71% during both the acute phase and after the acute course; in group 2, it was 63% during the acute phase and 72% in the post-acute phase. Laboratory blood tests included whole blood morphology, C-reactive protein (CRP), procalcitonin (PCT), alanine aminotransaminase (ALT), aspartate transaminase (AST), BNP, NT-proBNP, and troponin I.

### Statistical Analysis

The normality of the variable distributions was tested using the Shapiro–Wilk test. The *t*-test, Cochran–Cox test, Mann–Whitney test, or Fisher’s exact test was used, where applicable, to compare the variables between the two groups. Spearman correlation analysis was used to assess correlations between the variables. Statistical analysis was performed using Statistica 13 by TIBCO. *p* < 0.05 was considered statistically significant.

## 3. Results

A total of 44 adolescents were included in the analysis. There were no in-hospital deaths in the presented group. The median hospitalization time was 9 (7–11) days.

Chest pain was the leading presenting symptom, reported by all patients (100%). Palpitations were noted in two individuals (5%), and one patient (2%) presented with syncope.

On admission, electrocardiographic abnormalities involving the ST–T segment were observed in 25 patients (57%), including T-wave flattening or inversion, ST elevation, and ST depression. Transthoracic ECHO revealed a median (Q1-3) values of LVEF of 60% (63–67%).

Admission laboratory results are summarized in Table 1.

All patients underwent transthoracic ECHO both during the acute phase and in the post-acute period. Z-scores were assessed where appropriate. A statistically significant correlation (*p* < 0.001) was observed in LVEF between the two predefined subgroups, although no patient demonstrated severe systolic impairment. Twenty-four-hour Holter monitoring revealed supraventricular premature beats in 9 patients (20%), ventricular premature beats in 7 patients (16%), ventricular pairs in 3 patients (7%), and one patient (2%) was diagnosed with non-sustained VT. 

Detailed information is presented in Table 2. 

**Figure 1:**
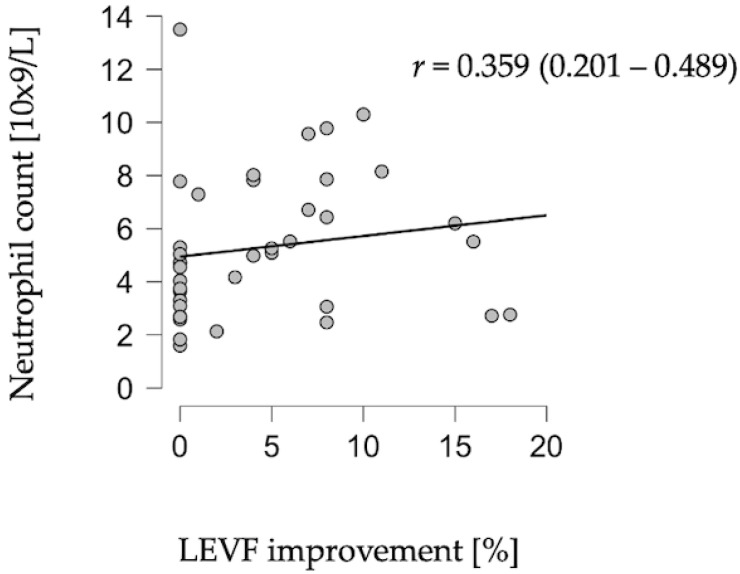
Correlation between neutrophil count and LVEF improvement.

A statistically significant correlation was identified between neutrophil count on admission and improvement in LVEF in the post-acute phase (r = 0.348, *p* = 0.028) (Figure 1).

No significant associations were found between LVEF and leukocyte count (*p* = 0.391), lymphocyte count (*p* = 0.831), monocyte count (*p* = 0.445), or NLR (*p* = 0.137). Similarly, markers of myocardial injury and hemodynamic stress—troponin I (*p* = 0.715) and BNP (*p* = 0.907)—did not correlate with LVEF in the improvement period.

## 4. Discussion

The study demonstrates a significant correlation between neutrophil count at hospital admission and subsequent improvement of left ventricular systolic function in adolescents with non-fulminant acute myocarditis. Notably, neutrophil count did not correlate with established markers of myocardial injury or hemodynamic stress, including troponin I and NT-proBNP. This finding suggests that neutrophil count may not directly reflect the extent of myocardial necrosis but could be associated with early functional changes captured by echocardiography. Due to limited and heterogeneous CMR data, correlations with tissue-level inflammatory markers could not be reliably assessed.

The exclusion of patients with fulminant myocarditis, cardiac tamponade requiring intervention, or Kawasaki disease was intentional, as these clinical states are known to substantially alter the neutrophil count in peripheral blood [34]. This design allowed for a more targeted evaluation of inflammation limited to uncomplicated myocardial involvement.

The literature has traditionally emphasized the detrimental consequences of neutrophil activation. Elevated neutrophil count or increased NLR has been independently associated with worse prognosis in adult myocarditis and myocardial infarction, reflecting extensive myocardial necrosis and systemic inflammation [26,27,28,29].

These findings align with mechanistic data showing that neutrophil-related pathways—including alarmin release (S100A8/S100A9) and formation of NETs—contribute to myocardial injury and dysfunction in adult heart failure and myocarditis.

In contrast, our results suggest that in pediatric patients with preserved or mildly reduced LVEF, neutrophils may serve an age-related immunologic role. Pediatric immune responses are characterized by stronger localized innate activation and a higher proportion of naïve T cells, which may facilitate efficient viral clearance without triggering the broader systemic inflammation typical of adults. This developmental divergence may explain why neutrophils function as markers of adverse remodeling in adults, as shown by Cannata et al. [29], while in children they may instead accompany a more controlled and effective antiviral response.

A recently published multicenter study by Madaudo et al. [35] demonstrated that an admission NLR ≥ 4 is a simple marker for risk stratification, particularly in individuals presenting with preserved LVEF. These findings highlight the prognostic value of systemic inflammatory indices in myocarditis and offer an important context for interpreting our results. NLR was not associated with LVEF improvement in our adolescent population with non-fulminant disease; instead, the absolute neutrophil count correlated positively with functional improvement. This discrepancy likely reflects differences in immunopathological response between mild pediatric myocarditis and more severe or adult phenotypes, suggesting that neutrophil-based markers may have distinct prognostic significance depending on age and clinical presentation.

Pediatric immune responses differ substantially from those of adults. Children exhibit higher proportions of naïve T cells and demonstrate stronger local innate immune reactivity, features that may favor efficient viral clearance and controlled initiation of reparative pathways. Adults, conversely, demonstrate a more pronounced systemic interferon response, predisposing them to broader inflammatory activation and remodeling [36,37].

The present results are partially consistent with data indicating that appropriately regulated neutrophil recruitment promotes clearance of necrotic debris while facilitating downstream reparative processes [23]. Thus, a moderately elevated neutrophil count in non-fulminant myocarditis may reflect an effective, rather than pathological, immunologic response.

In adult populations, elevated neutrophil count and increased neutrophil-to-lymphocyte ratio have consistently been associated with adverse outcomes in myocarditis and other cardiovascular conditions, likely reflecting extensive myocardial injury and systemic inflammation. In contrast, the present findings in a pediatric cohort with preserved or mildly reduced systolic function suggest that the prognostic implications of neutrophil-related markers may differ depending on age and disease severity. This discrepancy may reflect developmental differences in immune responses rather than opposing biological effects.

Therefore, the observed association between higher neutrophil counts and improved LVEF may reflect a balanced immune response rather than pathogenic inflammation. The observed association between admission neutrophil count and subsequent improvement in left ventricular systolic function should be interpreted with caution. The present study does not provide mechanistic evidence for a reparative role of neutrophils in myocarditis. Rather, absolute neutrophil count may serve as a marker reflecting the magnitude or balance of the early innate immune response in adolescents with non-fulminant disease. Future prospective studies in larger pediatric populations are needed.

Based on previously published data NLR correlates with LVEF in pediatric patients with acute myocarditis and is a better predictor than biomarkers such as CRP, PCT, high-sensitivity troponin, BNP and NT-proBNP.

In contrast, our study did not find any relevant association between the monocyte-to-lymphocyte ratio (MLR), eosinophil-to-lymphocyte ratio (ELR), and basophil-to-lymphocyte ratio and echocardiographic, electrocardiographic, and clinical parameters. It may supports the notion that isolated neutrophil count may be a more sensitive indicator of myocardial improvement in this specific population.

Since myocarditis presents with a broad spectrum of clinical presentation in children, this disease remains a challenge in a diagnostic workup in clinical practice [18].

We believe that the novelty of our analysis is based on the inclusion of preserved/mildly deteriorated myocardial injury analysis in adolescents presenting with acute myocarditis. Despite its limitations, this study addresses an understudied subgroup of adolescents with non-fulminant myocarditis and preserved or mildly reduced systolic function, a population often excluded from larger myocarditis cohorts.

Besides various triggering etiologies of myocarditis, predisposing factors have also been reported [38]. The autoimmune defense directed against the myocardium is triggered, involving an innate response mediated by the activation of monocytes/macrophages, neutrophils, and eosinophils [39]. The neutrophil infiltration in the myocardial tissue in acute myocarditis was presented in Zhang et al.’s analysis [40]. Humoral response is claimed to play a crucial role in hemodynamic complications [41,42].

Given the heterogeneous clinical presentation of myocarditis in children and adolescents, the identification of accessible prognostic markers remains essential. The present study provides evidence that absolute neutrophil count may serve as a simple and clinically relevant marker associated with early systolic improvement in non-fulminant myocarditis.

From a clinical perspective, our findings suggest that a simple and widely available parameter such as absolute neutrophil count may help identify adolescents with non-fulminant myocarditis who are more likely to experience early improvement of left ventricular function. Because complete blood count is routinely obtained at presentation, neutrophil count could be easily integrated into initial risk stratification and used to tailor the intensity of ECHO follow-up. If confirmed in larger prospective cohorts, this approach may support more individualized management of pediatric patients with acute myocarditis.

### Study Limitations

This study has several important limitations. It is a single-center, retrospective analysis with a limited sample size, as the diagnosis is relatively rare and the population is unique. It restricts statistical power and precludes multivariable adjustment. The diagnosis of myocarditis was supported by multimodal clinical assessment; however, cardiac magnetic resonance imaging was not performed uniformly in all patients, limiting tissue-level correlation analyses. The absence of immunologic or molecular markers to support mechanistic interpretation is another limitation. The authors are concerned with perioperative outcomes, so the follow-up duration is limited due to a lack of long-term outcomes. Additionally, the study population consisted exclusively of adolescents with preserved or mildly reduced left ventricular ejection fraction; therefore, the results cannot be generalized to fulminant myocarditis or severe systolic dysfunction. Finally, the observational nature of the study does not allow causal inference, and the observed association may represent a marker of disease phenotype rather than a mechanistic effect.

## 5. Conclusions

In adolescents hospitalized with non-fulminant acute myocarditis and preserved or mildly reduced systolic function, absolute neutrophil count at admission was associated with subsequent improvement in left ventricular ejection fraction during hospitalization. However, given the retrospective nature of the study and the limited sample size, we cannot conclusively determine whether neutrophil activation plays a reparative role.

Given the exploratory design and limited sample size, these findings should be interpreted as hypothesis-generating. Larger prospective studies incorporating standardized cardiac magnetic resonance imaging and immunologic profiling are required to clarify the clinical relevance and biological basis of this association.

## Figures and Tables

**Table 1:** Patients’ demographic, clinical, and laboratory characteristics.

Parameters	Group 1 N = 18 Unchanged LVEF	Group 2 N = 26 LVEF Improvement	*p*
Demographical			
Sex (F (%)/M (%))	3 (17)/15 (83)	4 (15)/22 (85)	0.889
Age (years) (median, (Q1–Q3))	15 (13–17)	16 (14–17)	0.561
BMI (median, (Q1–Q3))	20 (20–25)	23 (20–25)	0.991
Hospitalization			
Days (median, (Q1–Q3))	9 (8–9)	9 (7–11)	0.726
Clinical—symptoms			
Chest pain (*n*, (%))	18 (100)	26 (100)	1.00
Syncope (*n*, (%))	0 (0)	1 (4)	1.00
Heart palpitations (*n*, (%))	1 (6)	1 (4)	1.00
Whole blood count analysis on admission			
WBC [K/uL] (median, (Q1–Q3))	7.2 (6.4–8.7)	7.6 (6.7–10.4)	0.391
Hgb [g/dL] (median, (Q1–Q3))	13.8 (12.7–14.9)	14.4 (13.2–15.5)	0.330
Hct [%] (median, (Q1–Q3))	40.1 (37.1–44.3)	40.7 (38.8–44.4)	0.627
MCV [fl] (median, (Q1–Q3))	83.9 (81.4–85.7)	82.9 (80.7–85.4)	0.516
MCH [pg] (median, (Q1–Q3))	29.0 (28.2–29.5)	29.0 (28.0–29.6)	0.933
MCHC [g/dL] (median, (Q1–Q3))	34.3 (33.8–25.1)	34.6 (33.9–35.4)	0.190
PLT [K/uL] (median, (Q1–Q3))	238.0 (188.8–262.0)	239.0 (223.0–270.5)	0.835
RDW [fl] (median, (Q1–Q3))	38.7 (37.0–40.1)	37.7 (37.0–39.7)	0.513
NEUTR# [K/uL] (median, (Q1–Q3))	3.9 (2.8–5.0)	5.5 (4.4–7.9)	0.030 *
MON# [K/uL] (median, (Q1–Q3))	0.8 (0.6–1.2)	1.0 (0.7–1.2)	0.446
LYM# [K/uL] (median, (Q1–Q3))	1.9 (1.6–2.0)	1.7 (1.5–2.3)	0.838
NLR (median, (Q1–Q3))	1.9 (1.5–3.8)	3.5 (2.4–4.1)	0.138
IG# [K/uL] (median, (Q1–Q3))	0.0 (0.0–0.0)	0.0 (0.0–0.0)	0.574
Myocardial injury markers			
Troponin I (median, (Q1–Q3))	6066 (1224–13,383)	6777 (1683–16,611)	0.722
Other laboratory tests:			
CRP [mg/dL] (median, (Q1–Q3))	1.8 (1.1–4.7)	2.7 (1.7–5.7)	0.314
PCT [ng/mL] (median, (Q1–Q3))	0.08 (0.06–0.13)	0.14 (0.07–0.25)	0.118
ALT [IU/L] (median, (Q1–Q3))	24 (16–27)	21 (16–33)	0.604
AST [IU/L] (median, (Q1–Q3))	58 (33–102)	48 (37–86)	0.828
BNP [pg/mL] (median, (Q1–Q3))	31.7 (11.7–40.0)	30.6 (10.5–60.4)	0.920
NTproBNP [pg/mL] (median, (Q1–Q3))	267 (174–414)	367 (194–837)	0.262

Abbreviations: WBC—white blood count, Hgb—hemoglobin, Hct—hematocrit, MCV—mean corpuscular volume, MCH—mean corpuscular hemoglobin, MCHC—mean corpuscular hemoglobin concentration, PLT—platelets, RDW—red blood cell distribution, NEUTR—neutrophils, MON—monocytes, LYM—lymphocytes, NLR—neutrophil-to-lymphocyte ratio, IG—immature granulocytes, CRP—C-reactive protein, PCT—procalcitonin, ALT—alanine transaminase, AST—aspartate aminotransferase, BNP—brain natriuretic peptide, NTproBNP—N-terminal prohormone of brain natriuretic peptide. * *p* < 0.05.

**Table 2:** Echocardiographic and electrocardiographic results in the presented groups.

Parameters	Group 1 N = 18 Unchanged LVEF	Group 2 N = 26 LVEF Improvement	*p*
Echocardiographic results in an acute phase			
LVEF (%) (median, (Q1–Q3))	71 (67–71)	63 (60–67)	<0.001
LVED z-score (median, (Q1–Q3))	0.10 (−0.59–0.65)	0.61 (0.05–1.13)	0.102
RVED z-score (median, (Q1–Q3))	0.48 (0.04–0.83)	0.84 (−0.28–1.70)	0.339
IVs z-score (median, (Q1–Q3))	0.21 (−0.18–0.83)	0.56 (−0.24–1.45)	0.566
PWd z-score (median, (Q1–Q3))	0.09 (−0.08–0.85)	0.60 (−0.07–1.27)	0.319
Pericardial effusion [mm](median, (Q1–Q3))	0 (0)	0 (0)	0.780
E/A	1.7 (1.5–1.8)	1.8 (1.6–1.9)	0.440
E/E′	5.3 (4.8–6.4)	6.0 (5.2–7.1)	0.279
Echocardiographic results after acute course			
LVEF (%) (median, (Q1–Q3))	71 (69–74)	72 (69–74)	0.561
LVED z-score (median, (Q1–Q3))	0.15 (−0.60–0.61)	0.80 (−0.15–1.34)	0.070
RVED z-score (median, (Q1–Q3))	0.75 (0.40–1.2)	0.55 (0.14–1.17)	0.936
IVs z-score (median, (Q1–Q3))	0.00 (−0.63–0.39)	0.55 (−0.24–1.23)	0.110
PWd z-score (median, (Q1–Q3))	0.46 (−0.14–0.60)	0.21 (−0.07–0.60)	0.988
Pericardial effusion (mm)(median, (Q1–Q3))	0 (0)	0 (0)	0.970
E/A	1.8 (1.6–1.9)	1.7 (1.5–1.8)	0.446
E/E′	5.8 (5.0–6.6)	4.8 (4.5–5.5)	0.164
ST-T segment changes			
in II, III, aVF leads (*n*, (%))	12 (67)	12 (46)	0.285
in I leads (*n*, (%))	2 (11)	7 (27)	0.168
in V4 leads (*n*, (%))	3 (17)	14 (54)	0.057
in V5 leads (*n*, (%))	7 (39)	20 (77)	0.071
in V6 leads (*n*, (%))	7 (39)	18 (70)	0.134
Holter results			
Abnormalities	0 (0)	2 (87)	0.505
SVPB (*n*, (%))	2 (11)	7 (27)	0.27
VPB (*n*, (%))	2 (11)	5 (19)	0.682
VPB pairs (*n*, (%))	1 (6)	2 (8)	1.000
nsVT (*n*, (%))	1 (6)	0 (0)	0.409

Abbreviations: LVEF—left ventricular ejection fraction, LVED—left ventricular end-diastolic diameter, RVED—right ventricular end-diastolic diameter, IVs—interventricular septum, PWd—posterior wall diameter, SVPB—supraventricular premature, VPB—ventricular premature beats, nsVT—non-sustained VT.

## Data Availability

The data supporting the reported results can be obtained by contacting the corresponding authors within 3 years following publication, upon reasonable request.

## Abbreviations

The following abbreviations are used in this manuscript:

ALT	alanine transaminase
AST	aspartate aminotransferase
BNP	brain natriuretic peptide
CMR	cardiac magnetic resonance
CMV	cytomegalovirus
CRP	C-reactive protein
EBV	Epstein–Barr virus
ECG	electrocardiogram
ECHO	echocardiography
ELR	eosinophil-to-lymphocyte ratio
Hgb	hemoglobin
Hct	hematocrit
HCV	hepatitis C virus
HHV-6	human herpesvirus 6
HIV	human immunodeficiency virus
IG	immature granulocytes
IVs	interventricular septum
LYM	lymphocytes
LVED	left ventricular end-diastolic diameter
LVEF	left ventricular ejection fraction
MCH	mean corpuscular hemoglobin
MCHC	mean corpuscular hemoglobin concentration
MCV	mean corpuscular volume
MLR	monocyte-to-lymphocyte ratio
MON	monocytes
NLR	neutrophil-to-lymphocyte ratio
NT-proBNP	N-terminal prohormone of brain natriuretic peptide
NEUTR	neutrophils
nsVT	non-sustained ventricular tachycardia
PCT	procalcitonin
PWd	posterior wall diameter
RDW	red blood cell distribution width
RVED	right ventricular end-diastolic diameter
SARS-CoV	severe acute respiratory syndrome coronavirus
SVPB	supraventricular premature beats
VPB	ventricular premature beats
WBC	white blood cell count
